# Personalized circulating tumor DNA analysis for sensitive disease monitoring and detection of relapse in neuroblastoma

**DOI:** 10.1186/s40364-024-00688-5

**Published:** 2024-11-26

**Authors:** Ida Rahmqvist, Enya Engström, Elisabeth Mellström, Raghda R. Ibrahim, Fani Pujol-Calderón, Agnes Dahlstrand Rudin, Anna Ordqvist Redfors, Niki Rostamzadeh, Rita Di Rienzo, Wilma Franssila, Robert Khashan, Moe Xylander, Christin Karlsson, Torben Ek, Daniel Andersson, Tobias Österlund, Jennie Gaarder, Henrik Fagman, Susanne Fransson, Tommy Martinsson, Anders Ståhlberg, Martin Dalin

**Affiliations:** 1 Sahlgrenska Center for Cancer Research, Department of Pediatrics, Institute of Clinical Sciences, Sahlgrenska Academy at University of Gothenburg, Gothenburg, Sweden; 2 Wallenberg Centre for Molecular and Translational Medicine, University of Gothenburg, Gothenburg, Sweden; 3 Department of Physiology and Pharmacology, Karolinska Institutet, Stockholm, Sweden; 4 Children’s Cancer Cancer Centre, Queen Silvia Children’s Hospital, Sahlgrenska University Hospital, Gothenburg, Sweden; 5 Department of Pediatrics, Queen Silvia Children’s Hospital, Sahlgrenska University Hospital, Gothenburg, Sweden; 6 Sahlgrenska Center for Cancer Research, Department of Laboratory Medicine, Institute of Biomedicine, Sahlgrenska Academy at University of Gothenburg, Gothenburg, Sweden; 7 Department of Clinical Genetics and Genomics, Sahlgrenska University Hospital, Region Västra Götaland, Gothenburg, Sweden; 8 Department of Laboratory Medicine, Institute of Biomedicine, Sahlgrenska Academy at University of Gothenburg, Gothenburg, Sweden; 9 Department of Clinical Pathology, Sahlgrenska University Hospital, Gothenburg, Sweden

**Keywords:** Circulating tumor DNA, Neuroblastoma, Pediatric cancer, Liquid biopsy, Personalized medicine

## Abstract

Circulating tumor DNA (ctDNA) has shown potential as a non-invasive tumor biomarker in neuroblastoma. Previous studies used generic assays for detection of selected predefined oncogenic variants as markers of ctDNA, which limits the sensitivity and excludes a subset of patients from analysis. Here we assessed patient-specific ctDNA analysis for treatment evaluation and detection of relapse in neuroblastoma. We generated personalized sequencing panels targeting 10 tumor-specific single nucleotide variants (SNVs) for each patient and performed ctDNA analysis of 136 plasma samples collected longitudinally in 13 children with neuroblastoma. ctDNA was detected using ultra-deep next generation sequencing with unique molecular identifiers to eliminate polymerase-induced errors. We found that the levels of ctDNA at diagnosis correlated with risk group and decreased gradually during effective treatment. All samples collected during follow-up in patients without disease relapse were ctDNA-negative. All four relapses were associated with elevated ctDNA levels, and a majority of the analyzed SNVs were detected at time of relapse. In one case, ctDNA became positive 78 days before the relapse was diagnosed with routine assessment and increased by over a thousandfold before the start of additional treatment. Overall, ctDNA was more uniformly elevated at diagnosis, showed less putative false positive results, and was more sensitive for detection of relapse compared to five serum or urine tumor markers used in clinical routine. In conclusion, personalized ctDNA analysis is suitable for clinical monitoring of tumor burden and may be used in all neuroblastoma patients regardless of risk group or tumor genetics.

**Supplementary Information:**

The online version contains supplementary material available at 10.1186/s40364-024-00688-5.

To the editor

Neuroblastoma originates from the developing sympathetic nervous system and has variable prognosis depending on stage, tumor genetics and age at diagnosis [1]. The disease is mainly monitored with radiologic examinations and ^123^I-metaiodobenzylguanidine scintigraphy [2], which expose the patients to anesthetics and/or ionizing radiation and may be inconclusive in patients with low disease burden [3, 4]. Previous studies have evaluated ctDNA as a non-invasive biomarker in neuroblastoma by analyzing selected genetic alterations such as *MYCN* amplification or *ALK* mutations, which is only applicable in patients harboring those variants [5–8]. Others have used large generic next-generation sequencing panels, limiting the accuracy of the analysis at low levels of ctDNA [9–11].

**Fig. 1:**
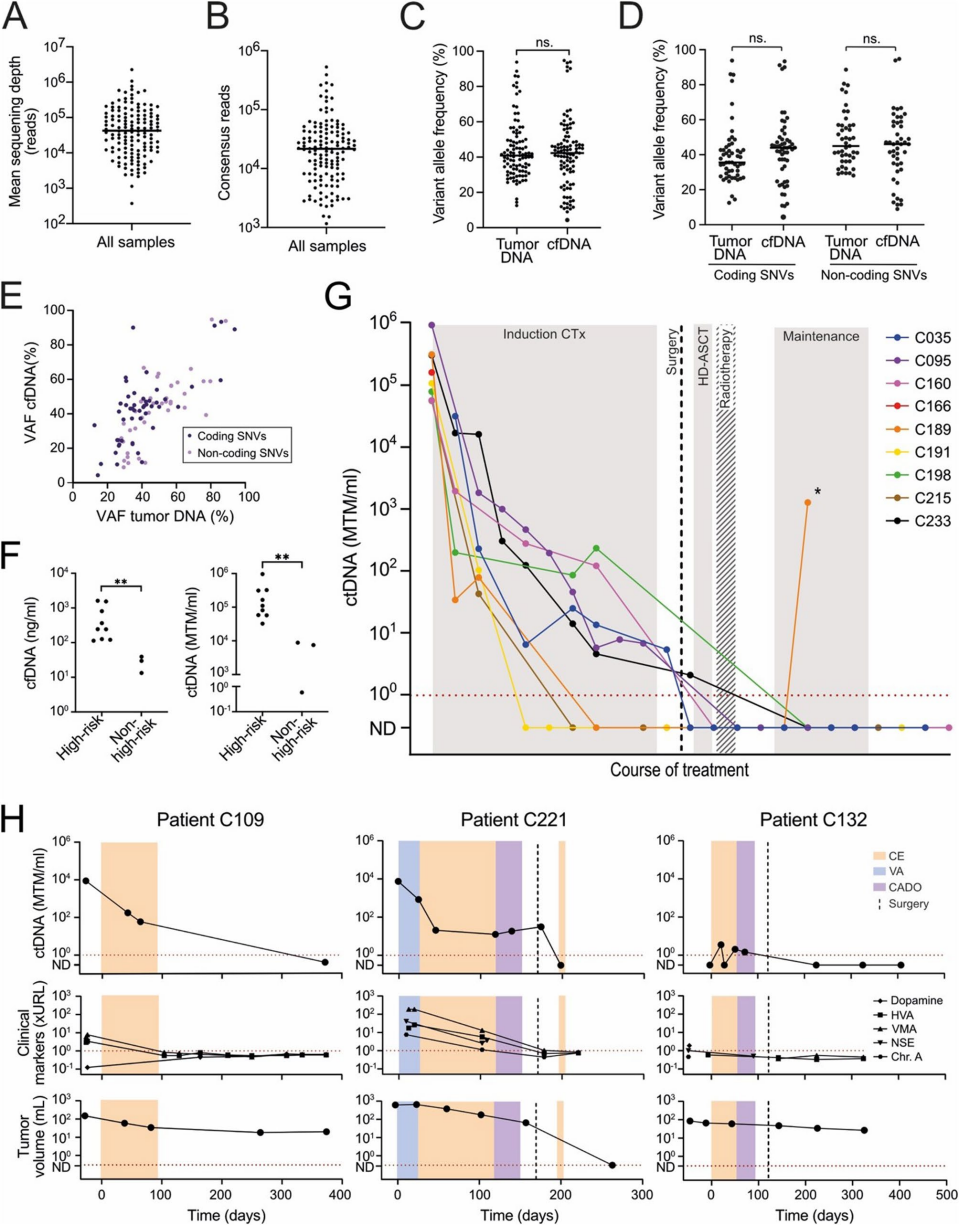
Personalized ctDNA analysis for monitoring tumor burden in neuroblastoma. **(A)** Sequencing depth. Each datapoint represents the mean sequencing depth for one plasma sample. Median, 42,180. **(B)** Number of consensus reads in all 10 assays combined per plasma sample. Median, 21,642. **(C)** Variant allele frequency in whole genome sequencing of tumor biopsy DNA versus SiMSen-seq of cfDNA at time of diagnosis. Median 40.9 vs. 42.3%. *P* = 0.06, Wilcoxon matched-pairs signed rank test. **(D)** Variant allele frequency in whole genome sequencing of tumor biopsy DNA versus SiMSen-seq of cfDNA at time of diagnosis for coding and non-coding SNVs, respectively. Median 35.6 vs. 44.0% (*P* = 0.16) for coding SNVs; 45.0 vs. 46.0% (*P* = 0.051) for non-coding SNVs. *P* value, Wilcoxon matched-pairs signed rank test. **(E)** Correlation between variant allele frequency in cfDNA at diagnosis and tumor biopsy DNA for coding and non-coding SNVs. *P* < 0.0001 for both coding and non-coding SNVs, non-parametric Spearman correlation test. Patients C035 and C125 were excluded from the analysis since no pre-treatment cfDNA sample was available from these patients. Patient C132 was excluded from the analysis since ctDNA was under the limit of detection at diagnosis. **(F)** Levels of cfDNA and ctDNA at time of diagnosis in children with HR and non–HR neuroblastoma. *P*-value (A and B) = 0.009, Two-tailed Mann Whitney test. MTM, mutated tumor molecules. **(G)** Total levels of ctDNA throughout treatment in patients with HR neuroblastoma. CTx, chemotherapy; HD-ASCT, high dose chemotherapy with autologous stem cell transplantation. ND, not detected. The asterisk denotes disease relapse. **(H)** Levels of ctDNA, clinical tumor markers, and approximated tumor volume over time in patients with low- or intermediate-risk neuroblastoma. For patient C109, approximated tumor volume was based on CT in the first two timepoints and MRI in the last three timepoints. For patient C221, approximated tumor volume was based on CT in timepoint two and six, and MRI in all other timepoints. For patient C132, approximated tumor volume was based on CT in the first three timepoints and MRI in last three timepoints. CE, carboplatin-etoposide; VA, vincristine-actinomycin D; CADO, cyclophosphamide-vincristine-doxorubicin; Chr. A, chromogranin A; ND, not detected

**Fig. 2:**
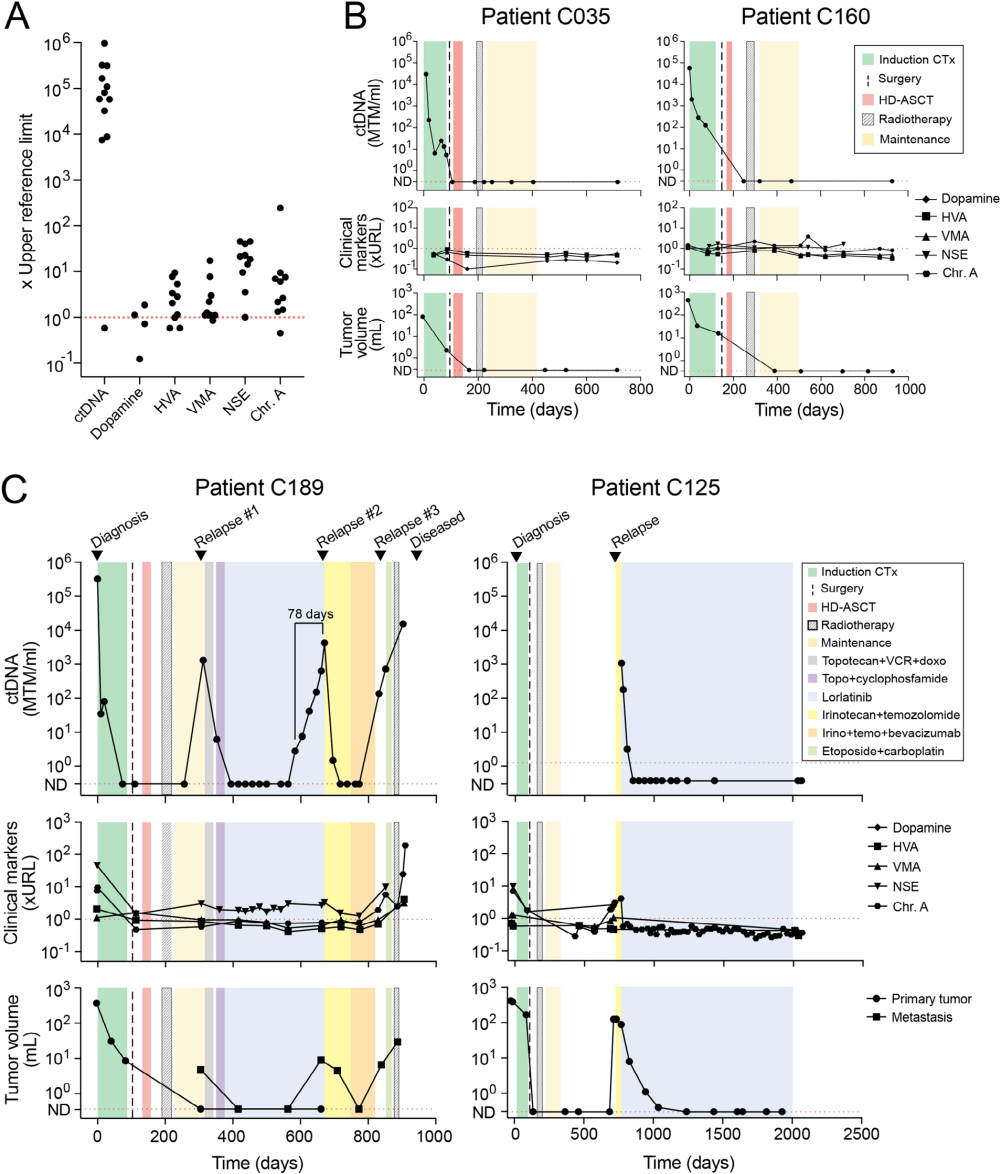
Levels of ctDNA compared to conventional tumor markers for evaluation of treatment response and detection of relapse. **(A)** Levels of ctDNA and clinical tumor markers at time of diagnosis. All values were normalized to upper reference limit, which for ctDNA was set to 1 MTM/ml. Chr. A, chromogranin A. **(B)** Levels of ctDNA, clinical tumor markers, and approximated tumor volume over time in patients C035 and C160. For patient C035, approximated tumor volume was based on CT in the first timepoint and MRI in all other timepoints. For patient C160, approximated tumor volume was based on CT in the first five timepoints and MRI in the last three timepoints. CTx, chemotherapy; HD-ASCT, high dose chemotherapy with autologous stem cell transplantation; MTM, mutated tumor molecules; ND, not detected. **(C)**. Levels of ctDNA, clinical tumor markers, and approximated tumor volume over time in patients C189 and C125. For patient C189, approximated tumor volume was based on ultrasound in timepoint two, MRI in timepoint nine and ten, and CT in all other timepoints. For patient C125, approximated tumor volume was based on CT in all timepoints. CTx, chemotherapy; HD-ASCT, high dose chemotherapy with autologous stem cell transplantation; doxo, doxorubicine; VCR, vincristine; Chr. A, chromogranin A. URL, upper reference limit; ND, not detected

We designed personalized ctDNA panels for detection of 10 SNVs per patient selected based on variant allele frequency (VAF) in whole genome sequencing of tumor and leukocyte DNA in 13 children with neuroblastoma (Fig. S1 and Supplementary information: Materials and Methods). We included 74 coding and 55 non-coding SNVs in the sequencing panels (Fig. S2). Six of the patients (46%) did not harbor *MYCN* or *ALK* aberrations and would therefore have been excluded from previous ctDNA studies focusing on those variants (Table S1). Cell-free DNA (cfDNA) from 136 plasma samples was analyzed with the personalized sequencing panels using *Simple multiplexed PCR-based barcoding of DNA for ultrasensitive mutation detection by next-generation sequencing* (SiMSen-Seq) (Supplementary data 1–3) [12]. During SiMSen-Seq, a unique molecular identifier (UMI) sequence is added to each DNA molecule. After PCR amplification and sequencing, we generated error-corrected consensus reads requiring a UMI family size of three or more. A consensus read positive for a tumor-specific SNV was considered a mutated tumor molecule (MTM), and the level of ctDNA was defined as the total number of MTM per milliliter of plasma for all 10 assays combined. The median sequencing depth was 42,180 reads (Fig. 1A), and the median total number of consensus reads was 21,642 per plasma sample (Fig. 1B). The SNVs had similar VAF in cfDNA at diagnosis as in tumor DNA, and the VAF in cell-free and tumor DNA correlated with each other both for coding and non-coding SNVs (Fig. 1C–E). High-risk (HR) patients presented with higher levels of cfDNA and ctDNA at time of diagnosis compared to non-HR patients (Fig. 1F).

Longitudinal analysis of HR neuroblastoma patients showed a stepwise decline of ctDNA during induction chemotherapy, which was more profound compared to the reduction in tumor volume (Fig. 1G, Fig. S3). The levels of ctDNA decreased gradually during treatment also in the non-HR patients (Fig. 1H). Patient C132 had a ganglioneuroblastoma, which is a mixed tumor type with features of both neuroblastoma and the benign counterpart ganglioneuroma. The tumor regressed spontaneously before the start of treatment, showed a marginal response to chemotherapy, and did not relapse during four years of follow-up. In line with a less malignant disease, ctDNA was undetectable at diagnosis, alternated between low and negative levels during neoadjuvant treatment, and was negative during follow-up in this patient.

In the nine patients who were alive without relapse at the latest follow-up, all 23 samples collected during maintenance therapy or after the end of treatment were ctDNA-negative. Five of these patients had remaining tumors visible on radiologic examinations after treatment, suggesting that ctDNA is undetectable in patients with non-malignant tumor rests (Table S2).

Serum NSE and chromogranin A, and urine dopamine, HVA and VMA were analyzed longitudinally as clinical routine. At diagnosis, these tumor markers were similar in HR and non-HR patients (Fig. S4) and varied between normal levels and up to ∼100 times the upper reference limit (URL). In contrast, all patients except one had ctDNA levels between 10^4^ and 10^6^ times URL, which was set to 1 MTM/ml (Fig. 2A). ctDNA correlated with the clinical course of disease in all patients (Fig. S5–S10 and Supplementary information: Clinical case summaries) and was informative also in those with inconclusive clinical biomarkers, such as C035 and C160 (Fig. 2B).

Two patients experienced a total of four relapses during the study (Fig. 2C). Patient C189 had a HR neuroblastoma harboring *ALK* p.F1174L. During maintenance therapy, the patient had a metastatic relapse which was treated with the ALK inhibitor lorlatinib. Two more relapses occurred during lorlatinib and after additional chemotherapy. All three relapses were associated with elevated levels of ctDNA. At the second relapse, six consecutive samples had gradually increased levels of ctDNA starting 78 days before the metastasis was discovered whereas the clinical biomarkers remained unaffected. When relapse treatment was started, the level of ctDNA had increased approximately 1,500 times since the first positive sample. Most of the SNVs analyzed were detected at all three relapses (Fig. S11A). Patient C125 had metastasized neuroblastoma positive for *ALK* p.R1275Q and was enrolled in the study at time of a metastatic relapse. The patient then received lorlatinib, which resulted in complete response and a stepwise reduction of ctDNA which became consistently negative after 84 days of treatment. The sequencing panel for patient C125 was based on the initial diagnostic biopsy collected two years prior to enrollment in the study. At time of relapse, 9 of 10 SNVs were detected in the cfDNA (Fig. S11B).

Taken together, our results suggest that personalized ctDNA analysis provide a clinically useful biomarker in children with neuroblastoma regardless of risk group and tumor genetics (Supplementary information: Discussion, limitations and future directions). As large-scale sequencing of tumor DNA becomes increasingly available, this method could be of value also in other malignancies.

## Electronic supplementary material

Below is the link to the electronic supplementary material.

Supplementary Material 1

Supplementary Material 2

Supplementary Material 3

Supplementary Material 4

## Data Availability

The datasets used and/or analysed during the current study are available from the corresponding author on reasonable request.

## Abbreviations

CT: Computed tomography
cfDNA: Cell-free DNA
ctDNA: Circulating tumor DNA
HR: High-risk
HVA: Homovanillic acid
INRG: International Neuroblastoma Risk Group
LINES: Low and intermediate risk neuroblastoma European study
MIBG: ^123^I-metaiodobenzylguanidine
MRI: Magnetic resonance imaging
MTM: Mutated tumor molecule
NSE: Neuron-specific enolase
SiMSen-Seq: Simple multiplexed PCR-based barcoding of DNA for ultrasensitive mutation detection by next-generation sequencing
SNV: Single nucleotide variant
SU: Sahlgrenska University Hospital
URL: Upper reference limit
VAF: Variant allele frequency
VMA: Vanillylmandelic acid
