# Supplementary material for: Personalized circulating tumor DNA analysis for sensitive disease monitoring and detection of relapse in neuroblastoma
Source: Biomark Res. 2024 Nov 26;12:148. doi: 10.1186/s40364-024-00688-5 (PMC11600567; doi:10.1186/s40364-024-00688-5)
Supplement: Supplementary file 4 — Supplementary Material 4 [file 40364_2024_688_MOESM4_ESM.docx]

**Supplementary information**

**Personalized circulating tumor DNA analysis for sensitive disease**

**monitoring and detection of relapse in neuroblastoma**

Rahmqvist and Engström et al.

**Materials and methods**

*Patients*

Sixteen patients diagnosed with neuroblastoma at Sahlgrenska University Hospital (SU), Gothenburg, Sweden, between April 2018 and November 2021, were enrolled in the study. Fifteen patients were enrolled at initial diagnosis and one was enrolled at time of disease relapse. Three patients were excluded from ctDNA analysis; two due to insufficient quality of tumor biopsy DNA excluding them from whole genome sequencing, and one due to lack of available blood samples. Written informed consent was signed by all legal guardians prior to inclusion. The study was approved by the regional ethical review board in Gothenburg (Ref. No. 655-17) with an amendment approved by the Swedish ethical review authority (Ref. No. 2019-06285). Disease stage and risk group were defined according to the International Neuroblastoma Risk Group (INRG) Staging System and the INRG classification, respectively (1). HR patients were treated according to the High-Risk Neuroblastoma Study 1.5 of SIOP-Europe (HR-NBL1/SIOPEN protocol, ClinicalTrials.gov: NCT01704716). Low- and intermediate risk patients were treated according to the Low and intermediate risk neuroblastoma European study (LINES, ClinicalTrials.gov: NCT01728155). Clinical data were collected from the medical records. In two patients (C125 and C189), separate plasma samples collected during parts of the treatments were previously analyzed using an *ALK* tyrosine kinase domain-specific sequencing panel (2).

*Blood sampling and plasma preparation*

Approximately 8.5 ml of blood was collected in cf-DNA/cf-RNA Preservative Tubes (Norgen Biotech). The samples were collected prior to chemotherapy infusion to avoid variation in ctDNA levels due to recent or ongoing medication. During active treatment, the patients’ central venous catheter or port-a-cath were used for the blood sampling. After the end of treatment when the patients were generally lacking a central line, samples were collected through peripheral vein puncture which was coordinated with clinical routine blood sampling. After up to seven days of storage in room temperature, the blood samples were centrifuged for 20 minutes at 420 x g. (Heraeus Megafuge 8R, ThermoScientific). Plasma was transferred to XLX2000-2D Biobanking tubes (LVL Technologies) using the Freedom EVO liquid handling robot (Tecan), and was stored in -80 °C.

*Extraction of cell-free DNA*

Plasma was thawed to room temperature in a water bath and centrifuged for 10 minutes at 16.000 x g. cfDNA was extracted from approximately 4 ml of plasma using QIAamp Circulating Nucleic Acid Kit (Qiagen). The cfDNA was quantified with Qubit dsDNA HS Assay Kit (ThermoFisher Scientific). When needed, cfDNA was concentrated using Vivacon 500 (Sartorius) with a molecular weight cutoff of 30 kDa, to a volume of 10–14 µL. An inhibition test was performed with quantitative PCR, using genomic DNA (Sigma-Aldrich) as positive control (3).

*Tumor and germline DNA sequencing*

Tumor DNA from frozen diagnostic biopsy tissue and germline DNA from the cell fraction of a blood sample were extracted using DNeasy blood and tissue kit (Qiagen). Libraries were prepared using TruSeq DNA PCR-Free library prep kit (Illumina), with 1 μg DNA input. Whole genome sequencing was performed at Clinical Genomics, SciLife Laboratories, Stockholm, Sweden, using a NovaSeq (Illumina) aiming for a coverage of 90x and 30x for tumor and germline DNA, respectively. Mapping of reads to the reference genome, removal of duplicate reads, and variant calling was performed using the Sentieon’s suite of bioinformatics tools (Sentieon Inc.). SNVs with an alternative variant read depth of ≥10 and a variant allele frequency of ≥10% that were absent in germline DNA and passed manual review using Integrative Genomic Viewer (https://igv.org) were considered valid.

*Patient-specific ctDNA panel design*

For each patient, a multiplex PCR panel was designed for analysis of 10 SNVs, selected based on variant allele frequency in the whole genome sequencing data. Variants in coding regions were prioritized, but non-coding SNVs were also included in cases with less than 10 valid exonic variants. PCR primers were designed using Primer-BLAST (4) or Panelplex (DNA Software), with a 60 °C annealing temperature and amplicon sizes raging from 75 to 105 bp. To minimize polymerase-induced errors in sequencing and amplification and to allow for accurate quantification of ctDNA at low concentration, we used “Simple multiplexed PCR-based barcoding of DNA for ultrasensitive mutation detection by next-generation sequencing” (SiMSen-Seq) (5). Thus, universal SiMSen-Seq sequences were added to the target primers, including a unique molecular identifier (UMI) sequence which enables correction for polymerase-induced errors and uneven amplification (6). SiMSen-Seq assays were evaluated in multiplex reactions as previously described (5).

*cfDNA library preparation and sequencing*

Construction of the SiMSen-Seq libraries was carried out in two PCR steps: barcoding PCR and adapter PCR. Barcoding PCR was performed in 15 ml, containing 1x Platinum SuperFi buffer, 0.2 Units Platinum SuperFi DNA polymerase (Thermo Fisher Scientific), 0.2 mM dNTP (Sigma-Aldrich), 40 nM of each SiMSen-Seq barcoding primer, 0.5 M L-carnitine inner salt (Sigma-Aldrich), and up to 20 ng of cfDNA. The temperature profile was 98 °C for 3 minutes followed by three cycles of amplification (98°C for 10 seconds, 60 °C for 6 minutes and 72 °C for 30 seconds), 65 °C for 15 minutes and 95°C for 15 minutes. In the beginning of the 65 °C step, 45 ng of protease (Streptomyces griseus, Sigma Aldrich) in 30 µl TE buffer pH 8.0 (Ambion, Thermo Fisher Scientific) was added to inactivate the DNA polymerase.

The adapter PCR was performed in 60 µl, containing 400 nM of each of the universal forward and reverse adapter primers (Integrated DNA Technologies) (5), 1×Q5 Hot Start High-Fidelity Master Mix (New England Biolabs), and 15 µl of barcoding PCR product. The temperature profile was 98 °C for three minutes followed by 27 amplification cycles (98 °C for 10 seconds, ramping from 80 °C for one second down to 72 °C for 30 seconds and up 76, 0.2 °C/second, 76 °C for 30 seconds). Libraries were assessed using a HS NGS Fragment Kit (Agilent Technologies) on a 5200 Fragment Analyzer (Agilent Technologies). The PCR product was purified using Pippin Prep (Sage Science) with the target range of 205–300 bp.

Sequencing of the library pool at a final concentration of 1.2–1.8 pM was performed with the MiniSeq or NextSeq 1000 system (Illumina), containing a 20% PhiX positive control (Illumina) and using single-end reads with 150 bp mode.

*cfDNA sequencing data analysis*

The cfDNA sequencing data was analyzed bioinformatically with UMIErrorCorrect as described previously (7). In brief, sequencing reads were aligned to the Human Build 38 reference genome and reads were grouped into UMI families based on target DNA regions. Error-corrected consensus reads were generated requiring a UMI family size of at least three. A mutated tumor molecule (MTM) was defined as a consensus read harboring a tumor-specific SNV, and the total number of MTM per milliliter of plasma determined the level of ctDNA. Since SiMSen-Seq generates a mean of two barcodes per DNA molecule, the concentration of original mutated DNA molecules is approximately half of the MTM value. At least 1 MTM/ml was required for a sample to be reported as ctDNA-positive. One assay in one of the patients (C189) failed and showed no consensus reads at any timepoint, rendering a panel of nine SNVs.

*Assessment of tumor volume*

Volumes of primary tumors were assessed assuming the volume of an ellipsoid (V=1/6 x π x d1 x d2 x d3). Information about tumor diameters in three dimensions (d1–d3) was retrieved from radiology reports of MRI, CT, or in a few cases ultrasonography examinations. In cases where patient records lacked appropriate radiologic measurements, such measurements were performed by a resident physician in pediatrics using XERO Universal Viewer (Agfa Healthcare).

In some cases, exact measurements of tumor diameters in all three dimensions were not available due to difficulties in delineating the tumor margins. Tumor volumes were then estimated as follows: 1) When information about tumor diameter in one of three dimensions was missing, the tumor size change was assumed to be proportionally similar in all three dimensions. 2) In lack of radiographs visualizing the craniocaudal expansion of the tumor, measurements were performed based on information on vertebral level tumor spread described by the radiologist. 3) For small tumors lacking diameter measurements in one of three dimensions, the unknown diameters were assumed to be the mean of the two measured diameters. 4) Small tumors lacking information about size of tumor diameter in two of three dimensions were assumed to be spherical.

*Analysis of clinical biomarkers*

The clinical biomarkers were analyzed at the Department of Clinical Chemistry, SU, as part of the clinical routine. Data are reported normalized to the upper reference limit (URL) value. NSE was analyzed in serum with electrochemiluminescence technology, URL 16.3 µg/L. Chromogranin A was analyzed in serum using immunofluorescence with TRACE technology, URL 102 µg/L. Dopamine, HVA and VMA were analyzed in urine using mass spectrometry. URL for U-dopamine was 2200 (age <2.5 years), 1100 (age 2.5–5 years), 800 (age 5–10 years), or 400 µmole/mole/creatinine (age >10 years). URL for U-HVA was 20 (age <2 years), 14 (age 2–5 years), 9.4 (age 5–10 years), or 7.9 mmole/mole/creatinine (age >10 years). URL for U-VMA was 10.7 (age <2 years), 6.3 (age 2–5 years), or 4.7 mmole/mole/creatinine (age >10 years).

*Statistical analysis*

Statistical analysis and graph visualization was performed in GraphPad Prism and RStudio. Statistical tests are indicated in the respective sections and figure captions. A p-value less than 0.05 was considered statistically significant and level of significance is indicated in the figures: ns ≥0.05, *< 0.05, **< 0.01, ***< 0.001.

*Data availability*

Data from the patient-specific sequencing panels is available in the Supplementary information. Whole genome sequencing of tumor and germline DNA was performed as part of the clinical diagnostic process and is not available to the public to protect the privacy of the patients.

**Clinical case summaries**

**Patient C035.** The patient presented with swelling of the right cheek at four months of age. A CT scan showed a soft-tissue tumor causing extensive destruction of the mandible. Further investigation revealed a primary neuroblastoma in the left adrenal gland measuring 7 x 4 x 4 cm. Genetic findings included *MYCN* amplification, chromosome 1p deletion and chromosome 17q gain. Tumor cells were seen in bone marrow biopsies. MIBG scintigraphy was negative. Due to rapid tumor growth, treatment with etoposide and carboplatin according to the LINES protocol was started prior to definitive diagnosis. As the complete pathology report from the biopsies became known, treatment was changed to the SIOPEN neuroblastoma HR protocol and induction chemotherapy with Rapid COJEC. Bone marrow biopsies after four courses of chemotherapy were free from tumor cells. MRI of the abdomen and mandible after completion of Rapid COJEC induction showed good response with the abdominal tumor measuring 1.5 cm. The patient underwent surgery with removal of the primary tumor. A small tumor located adjacent to the inferior vena cava had to be left in place. The patient was then treated with high-dose chemotherapy followed by autologous stem cell transplantation. After that, the patient received 21 Gray of proton beam radiotherapy directed at the primary tumor. Finally, the patient received maintenance therapy with alternating courses of vitamin A and dinutuximab. Prior to maintenance therapy an open biopsy of the right mandible and repeat bone marrow biopsies showed no tumor involvement. Follow-up with regular MRI abdomen and chest x-rays has been unremarkable. The patient has impaired hearing and kidney function but remains free from disease four years after the end of treatment.

**Patient C095.** The patient presented with a swelling in the right temporal region at 15 months of age. A CT scan showed bone destruction and a soft tissue tumor with intra- and extracranial components. Further investigation showed a primary neuroblastoma in the right adrenal gland measuring 9.5 x 4.0 cm, with suspected metastases in the liver and pancreas, enlarged iliac lymph nodes, suspected bilateral pleural metastases, and bone marrow involvement. Genetic evaluation showed *MYCN* amplification, chromosome 11q deletion, chromosome 1p deletion and *TERT* rearrangement. The patient was treated according to the SIOPEN neuroblastoma HR protocol. After completing Rapid COJEC induction chemotherapy, the patient received a single dose of cyclophosphamide to prepare for harvesting of autologous stem cells and thereafter two courses of topotecan, vincristine and doxorubicin (TVD) prior to surgery. After surgery the patient received high-dose chemotherapy with autologous stem cell transplantation (HD+ASCT), followed by 21 Gray of proton radiotherapy to the primary tumor region. Finally, the patient received maintenance therapy with alternating courses of vitamin A and dinutuximab. Post treatment evaluation showed no evidence of disease on chest CT, abdominal MRI, MIBG, or bone marrow biopsies. The patient passed away one month after the end of therapy due to a cerebral haemorrhage. The autopsy did not show brain metastasis.

**Patient C109.** The patient presented at 15 months of age with tremor and unsteady gait. Diagnostic investigations showed paraspinal neuroblastoma measuring 11 x 6 x 4 cm, extending from verterbra Th5 to L1. The tumor displayed a chromosome 2p gain and segmental chromosomal aberrations. However, no *MYCN* amplification, 11q deletion or 17q gain were found and the patient was free from metastases. The patient was treated for opsoclonus-myoclonus syndrome (OMS) with corticosteroids and intravenous immunoglobulin (IVIG) therapy starting two weeks before chemotherapy. The neuroblastoma was classified as low risk, stadium L2 and treatment according to the SIOPEN LINES protocol, treatment group 3 was started with courses of carboplatin and etoposide (CE). The OMS treatment was omitted during chemotherapy. MRI after two and four chemotherapy courses showed reduction in tumor size. As the tumor had responded to chemotherapy and surgery was deemed to be risky due to tumor involvement of spinal blood vessels, surgery was not attempted. The patient was followed up with monthly urine catecholamine metabolites and regular imaging of the residual tumor. Four months after the end of therapy the patient developed tremor and was restarted on OMS treatment with IVIG, dexamethasone and rituximab. At the latest follow-up 58 months after the end of chemotherapy, the patient remained well without any treatment.

**Patient C125.** The patient was diagnosed at nine years of age with a neuroblastoma in the left adrenal gland measuring 4 x 3 cm with a large (12 x 9 x 8 cm) metastasis adjacent to the pancreatic tail. Genetic analysis showed a chromosome 1p deletion and an *ALK* p.R1275Q mutation. The patient was stratified as stadium III and treated with Rapid COJEC induction chemotherapy, surgery, radiotherapy and vitamin A. Thirteen months after the end of treatment, a mediastinal tumor and bilateral lung metastases were detected. Needle biopsies confirmed a relapse of the neuroblastoma harboring *ALK* p.R1275Q. The patient received two courses of irinotecan and temozolamide, and then continued with the ALK inhibitor lorlatinib as monotherapy. The patient was enrolled in the study at time of relapse and the first plasma sample was collected before the start of lorlatinib treatment. A CT chest performed two months into treatment showed marked reduction in size of the mediastinal tumor, and the pulmonary metastases were no longer visible. The patient remained on lorlatinib for 40 months and has been monitored for 14 months after the end of treatment with no signs of disease.

**Patient C132.** The patient presented at 15 years of age with chest pain. Diagnostic investigations showed a paravertebral tumor in the posterior mediastinum measuring 9.5 x 9 x 6 cm. Needle biopsies revealed the tumor to be a ganglioneuroblastoma with multiple numerical and segmental chromosomal abnormalities. No evidence of metastases was found. Since the diagnostic process took long time, a second MRI was performed one month after detection of the tumor and showed spontaneous shrinkage of the tumor. The patient was treated according to the LINES protocol, study group seven. After two courses of carboplatin and etoposide (CE), CT showed only a marginal reduction in tumor size. The treatment continued with two courses of cyclophosphamide, vincristine, and doxorubicin (CADO). Surgery was performed with removal of approximately 95 percent of the tumor. Viable tumor growth was seen microscopically, with partially small cell tumor, mixed with areas of Schwann cell differentiation and neurinoma component. The patient received no further treatment due to low risk of relapse and remains in remission after four years of follow-up.

**Patient C160.** The patient was diagnosed with a tumor in the left adrenal gland during an abdominal ultrasound performed due to fever of unknown origin. Further investigation showed a neuroblastoma measuring 7 x 8 x12 cm with multiple skeletal metastases (in the right scapula, multiple vertebrae, and two ribs on the left side). The tumor displayed segmental chromosomal abnormalities including chromosome 1p deletion, chromosome 17q gain, and *MYCC* amplification. The patient was treated according to the SIOPEN neuroblastoma HR protocol. Assessment with CT scan after four courses of chemotherapy showed a good partial response. Induction therapy was followed by two courses of topotecan, vincristine and doxorubicin (TVD) with the goal of further reducing the tumor before surgery. The tumor measured 2.5 x 2.2 x 5.5 cm on a CT scan two weeks prior to surgery. At least 95% of the tumor was removed during surgery and microscopic examination of the excised tumor showed mostly necrotic material. The patient was then treated with high-dose chemotherapy with autologous stem cell transplantation (HD+ASCT), followed by 21 Gray of proton beam radiotherapy directed at the primary tumor. Finally, the patient received maintenance therapy with alternating courses of vitamin A and dinutuximab. A CT scan after two months of maintenance therapy and a full body MRI one year after completed therapy showed no tumors. In line with this, the patient remains free from disease two and a half years after the end of treatment. Pathological uptake on MIBG is indicated by red arrows.

**Patient C166.** The patient presented at two years of age with a few months history of fatigue, vomiting and weight loss. An abdominal ultrasound showed a tumor situated adjacent to the left kidney. On a subsequent CT scan the tumor measured 8.5 x. 9.5 x 14.5 cm and was seen to encase the abdominal aorta. Needle biopsies revealed the tumor to be a neuroblastoma. There were no signs of metastases. Genetically the tumor displayed segmental chromosomal abnormalities including 1p del, 2p gain and *MYCN* amplification. Treatment according to the SIOPEN neuroblastoma HR protocol was started with Rapid COJEC course A. The patient suffered from high blood pressure during chemotherapy and anti-hypertensive treatment was started. A few days after finishing the first course of chemotherapy, the patient went into circulatory failure with cardiac arrest and passed away.

**Patient C189.** The patient presented at two years of age with fever, lethargy, poor weight gain and a lump in front of the right ear. Diagnostic investigations revealed a neuroblastoma in the right adrenal gland with a metastasis in the right mandible and multiple metastases in the lungs and liver, as well as bone marrow involvement. The tumor displayed *MYCN* amplification, chromosome 1p deletion, chromosome 17q gain and an *ALK* p.F1174L mutation. MIBG showed pathological uptake in the tumor and in multiple metastases. The patient was treated according to the SIOPEN neuroblastoma HR protocol. Evaluation after four courses of chemotherapy showed size reduction of the primary tumor, only one liver lesion was visible, and the bone marrow biopsy was free from malignant cells. At the end of induction chemotherapy, the adrenal tumor had shrunk further, no lung metastases were seen and MIBG showed no pathological uptake. The patient underwent surgery and high-dose chemotherapy with autologous stem cell transplantation (HD+ASCT), followed by 21 Gray of proton beam radiotherapy directed at the primary tumor. After two months of maintenance therapy with vitamin A and dinutuximab, a soft-tissue tumor at the right angle of the jaw was discovered and a biopsy confirmed it to be a relapse with similar genetic alterations as in the primary tumor. After one course of topotecan, vincristine and doxorubicin (TVD) and one course of topotecan and cyclophosphamide (TC), the patient was treated with the oral ALK inhibitor lorlatinib as monotherapy. After four months of lorlatinib treatment MIBG showed reduced uptake, but a planned CT scan after ten months of treatment showed a new tumor at the right mandible. A biopsy of the lesion confirmed it to be a second neuroblastoma relapse. MIBG did not show signs of other metastases. Relapse chemotherapy with irinotecan and temozolamide was started and two courses into treatment the soft-tissue component of the tumor sowed size reduction on CT. Bevacizumab was added to irinotecan and temozolamide from the fifth course. After seven chemotherapy courses a new tumor protruded from the right mandible. The patient received a course of etoposide and carboplatin while waiting for a tracheostomy to prepare for radiotherapy under general anaesthesia. Two months after the start of radiotherapy (30 Gray against the mandibular metastasis), the patient passed away due to progressive disease.

**Patient C191.** The patient presented at two years of age with constipation, fatigue, weight loss, and a palpable abdominal mass. Diagnostic investigations revealed a retroperitoneal left sided neuroblastoma measuring 11 x 10 x 8 cm. No metastases were found but the presence of an *MYCN* amplification stratified the patient to treatment according to the SIOPEN neuroblastoma HR protocol. MRI after four courses of chemotherapy showed good partial response (tumor measuring 7x5x4 cm). Half of the seventh and the whole eight chemotherapy course was omitted due to kidney failure. Evaluation with MRI after induction chemotherapy showed further reduction in tumor size (6 x 2.5 x 2.5 cm). The patient underwent surgery with a macroscopically radical tumor excision. The patient´s kidney function improved after surgery and one course of topotecan, vincristine and doxorubicin (TVD) was given, but high-dose chemotherapy with autologous stem cell transplantation was omitted. Treatment was continued with 21 Gray of proton beam radiotherapy directed at the primary tumor followed by maintenance therapy with alternating courses of vitamin A and dinutuximab. MRI a few weeks into maintenance therapy showed a small residual tumor (2.0 x 0.9 x 0.4 cm), which was not visible on MRI after finalized maintenance therapy. The patient remains free from disease 2.5 years after the end of treatment.

**Patient C198.** The patient presented at two years of age with lethargy, weight loss and fever. Diagnostic investigations showed a para-aortal neuroblastoma measuring 10 x 4 cm with multiple skeletal metastases and bone marrow involvement. Tumor genetics showed deletions of chromosome 1p and 11q. The patient was treated according to the SIOPEN neuroblastoma HR protocol and evaluation after four courses of chemotherapy showed reduction in size of the abdominal tumor but persisting MIBG positivity and neuroblastoma cells in the bone marrow. Evaluation after all eight chemotherapy courses still showed partial response with MIBG positivity and small amount of neuroblastoma cells in the bone marrow. Treatment was continued with two courses of topotecan, vincristine and doxorubicin (TVD), after which no malignant cells could be seen in the bone marrow, but MIBG continuously showed uptake in the abdomen and in multiple skeletal locations. Surgery was performed with resection of four different tumor components, but post-surgery MRI showed residual tumors adjacent to both kidneys. The patient received high-dose chemotherapy with autologous stem cell transplantation (HD+ASCT) followed by radiotherapy with 21Gy towards the abdomen, and maintenance treatment with alternating courses of vitamin A and dinutuximab. MIBG four months after the end of treatment showed continuous uptake in abdominal lymph nodes. The patient remains free from relapse after two years of follow-up.

**Patient C215.** The patient presented at three years of age with lethargy, weight loss and night sweats. Diagnostic investigations showed a neuroblastoma in the right adrenal gland. The primary tumor infiltrated the liver and dislocated the right kidney and inferior vena cava and was metastasized to the iliac bone and bone marrow. Tumor genetics showed *MYCN* amplification, chromosome 1p deletion and whole chromosome 17 gain. The patient was treated according to the SIOPEN neuroblastoma HR protocol. Evaluation after four courses of chemotherapy showed reduction in tumor size and no malignant cells in the bone marrow. After completed induction therapy the tumor was further reduced and MIBG showed no pathological uptake. The patient received two courses of topotecan, vincristine and doxorubicin (TVD) followed by surgery. After high-dose chemotherapy with autologous stem cell transplantation (HD+ASCT), the patient developed veno-occlusive disease leading to liver dysfunction and pulmonary haemorrhage and was treated with extracorporeal membrane oxygenation (ECMO). Due to the liver complications, radiotherapy (21 Gray towards the primary tumor) was postponed and given in parallel with the maintenance therapy. MIBG after the end of treatment did not show any pathological uptake, and the patient remains free from relapse after 20 months of follow-up.

**Patient C221.** The patient presented at nine months of age with a palpable abdominal mass. MRI of the abdomen showed a tumor measuring 13 x 10 x 9 cm originating from the right kidney. As the imaging was highly suggestive of Wilms tumor, treatment was started according to the UMBRELLA protocol. However, the pathology report revealed the tumor to be a neuroblastoma. MIBG and bone marrow biopsy showed multiple skeletal metastases and malignant cells in the bone marrow. The tumor displayed chromosome 11q deletion, chromosome 2p gain, and chromosome 17q gain. The patient was stratified as intermediate risk and was treated according to study group 10 in the LINES protocol. After two courses of carboplatin and etoposide (CE) the tumor had shrunk by approximately 35% and after another two courses the tumor was estimated to approximately 50% of its original size. Further chemotherapy with two courses of cyclophosphamide, vincristine, and doxorubicin (CADO). Surgery was performed, and large parts of the excised tumor were assessed as mature ganglioneuroblastoma. Post-operative bone marrow biopsy was clear of neuroblastoma cells, but MIBG still showed a slight skeletal uptake, mainly in the left tibia. The patient therefore received one more course of CE. CT abdomen and MIBG three months after the end of treatment were unremarkable, and the patient remains free from disease after 26 months of follow-up.

**Patient C233.** The patient presented at six years of age with fever, night sweats and low blood cell counts. Diagnostic investigations showed a pre- and paravertebral neuroblastoma in the posterior mediastinum measuring 20 x 8 x 4 cm. with widespread metastasis in the liver and skeleton. The tumor displayed chromosome 1p deletion, chromosome 17q gain and chromosome 11q deletion. The patient was treated according to the SIOPEN neuroblastoma HR protocol. Evaluation after four courses of chemotherapy showed reduction in tumor size and sparse presence of neuroblastoma cells in the bone marrow and after completed induction chemotherapy, the primary tumor was further reduced, and the bone marrow was negative for neuroblastoma cells. MIBG before surgery showed no abnormal uptake in skeleton or bone marrow. The tumor could not be radically removed during surgery due to risk of damaging arteries supplying the spinal cord. The patient was given high-dose chemotherapy with autologous stem cell transplantation (HD+ASCT), followed by radiotherapy with 21Gy towards the abdomen and maintenance treatment with alternating courses of vitamin A and dinutuximab. MRI after the end of treatment showed a remaining mass at the site of the primary tumor, whereas MIBG during maintenance therapy was negative. The patient remains free from relapse after 17 months of follow-up.

**Discussion, limitations and future directions**

Although significant progress has been made in the development of ctDNA as a tumor marker in neuroblastoma, previous studies have focused on pre-defined genetic alterations which leads to exclusion of patients lacking the selected variants (8-14). Here, we show that personalized sequencing panels may be used for sensitive and specific analysis of ctDNA in all investigated patients with neuroblastoma.

We developed multiplexed sequencing panels for detection of 10 SNVs per patient. This theoretically improves the sensitivity as compared to analyzing a single genetic alteration, which reduces the need for large plasma volumes. In small children, enabling ctDNA analysis from low plasma volume is crucial since excessive blood sampling may cause anemia (15). The use of 10 SNVs was chosen arbitrarily, and further studies are needed to determine the optimal number of assays in the patient-specific panels.

Most ctDNA studies focus on genetic alterations that are known to be oncogenic drivers. We included putative passenger SNVs in genes not associated with cancer as well as non-coding variants with no known function in our patient-specific sequencing panels. Since only two of the panels included an established oncogenic mutation (*ALK* p.R1275Q in patient C125 and *ALK* p.F1174L in patient C189), we were not able to compare the sensitivity between driver and passenger variants as markers of ctDNA. However, our detection of ctDNA also at low tumor burden suggests that non-driving genetic alterations can be sensitive markers of ctDNA.

Amplification of *MYCN* is a common feature in HR neuroblastoma, and levels of circulating *MYCN* have been assessed as a marker of ctDNA (10, 16). In some cases, the number of *MYCN* copies have increased prior to clinical detection of relapse, which may suggest it to be as sensitive as personalized ctDNA analysis. Apart from being applicable in all neuroblastoma patients and not only the minority harboring *MYCN* amplification, there are other potential benefits with our method: 1) Using ten genetic alterations as markers of ctDNA instead of one reduces the risk of missing a relapse arising from a subclone lacking the selected alteration. 2) Using SNVs instead of gene amplifications as markers of ctDNA may be beneficial during follow-up, since the SNVs are not detectable at all in cancer-free patients. Normal *MYCN* is still present in plasma after the end of successful treatment, and it may be difficult to differentiate between a relapse of the disease and physiological fluctuation of *MYCN* levels. 3) Using the same method in all patients regardless of genetic alterations makes it easier to compare results between different patient groups and draw conclusions about the clinical value of ctDNA analysis.

Previous ctDNA studies have utilized targeted next generation sequencing panels designed to include multiple genes recurrently mutated in neuroblastoma, aiming to be applicable in as many patients as possible. Two recent studies made use of panels covering coding regions of 62–68 genes and detected genetic alterations as markers of ctDNA in 73–82% of children with HR neuroblastoma (12, 13). Despite having a total genomic footprint of 140 and 500 kb of DNA, respectively, only one or a few mutations were detected by these panels in most patients. With our patient-specific approach, we were able to sensitively analyze 10 SNVs with panels covering only ~1 kb of DNA.

The FoundationOne Liquid CDx platform is an FDA approved NGS panel which was used to monitor patients with ALK mutated neuroblastoma in a study by Berko et al. (14). The panel covers parts of 311 genes and has an established detection limit of approximately 0.3-0.5% in variant allele frequency for different SNVs (17). Since our patient-specific panels resulted in a median of 22,000 error-corrected consensus reads and we required 4 mutated reads (1 MTM/ml of plasma in cfDNA from 4 ml of plasma), our limit of detection was approximately 0.018% which is approximately 20 times lower than that of FoundationOne Liquid CDx. In total, 22 of the 66 ctDNA-positive samples in our study showed levels below the detection limit of FoundationOne Liquid CDx. The high sensitivity of our method did not seem to come at the cost of low specificity, since all 23 samples collected during maintenance treatment or follow-up in patients without relapse were ctDNA negative.

Generating personalized sequencing panels is more time- and resource consuming compared to using the same targeted sequencing panels for all patients, but is likely conferring higher sensitivity and specificity for ctDNA analysis as a marker for tumor burden. On the other hand, our tumor DNA-guided approach is not optimal for determining mechanisms of drug resistance, which has been done effectively with larger NGS panels (14).

A limitation of our approach is that only somatic SNVs detected in the primary tumor are included in the personalized sequencing panel. Previous studies have shown discordance between genetic alterations in tumor DNA versus cfDNA collected at the same time, and subclonal driving alterations not detected in the tumor biopsy may still be present in other parts of the primary tumor or in metastatic sites (12). Targeting several SNVs may reduce the risk for the assay to miss a relapse or development of treatment resistance. In our cohort, a majority of the SNVs were detected at time of relapse in all four occurrences.

The levels of cfDNA and ctDNA at diagnosis were significantly higher in HR than in non-HR patients. Two patients (C166 and C191) with localized disease who were classified as HR due to genetic risk factors had ctDNA levels comparable with HR patients with metastasized disease. On the other hand, one patient (C221) who had widely metastasized disease but was classified as IR due to age less than one year at diagnosis and lack of risk-stratifying genetic alterations had ctDNA levels comparable to IR patients with localized disease. This suggests that ctDNA levels at diagnosis are determined by tumor biology rather than disease stage in children with neuroblastoma.

In most patients, the levels of ctDNA showed a stepwise reduction during induction chemotherapy, as evident by a relatively straight line in the logarithmic graphs. This is consistent with the log-kill hypothesis, stating that a given dose of chemotherapy kills a similar fraction of cells regardless of tumor size at the time of treatment (18). The decline in ctDNA levels during induction chemotherapy in HR patients was more pronounced than the reduction in tumor volume based on radiology examinations. Since tumors generally show a reduced density of viable cells following chemotherapy, this supports the hypothesis that ctDNA is a surrogate marker for total burden of malignant cells rather than tumor size. Personalized ctDNA analysis may therefore be an informative complement to imaging for evaluation of treatment. In line with this, the five patients in our cohort who had non-progressing residual tumors after the end of treatment were all ctDNA-negative.

The personalized ctDNA analysis showed several benefits compared to five different tumor markers used in the clinic (NSE and chromogranin A in plasma; dopamine, HVA and VMA in urine). First, whereas the clinical tumor markers at time of diagnosis were close to normal in many cases and 10 to 100 times elevated at most, ctDNA levels were generally between 10^4^ and 10^6^ times the level of detection. The wide range between high and low ctDNA concentrations enables interpretation of changes at different phases of the treatment. Second, high levels of ctDNA were detected at all four relapses, whereas most of the clinical markers were unaffected. Finally, one or more of the clinical biomarkers remained mildly elevated for long time after successful treatment in some cases, leading the clinicians to schedule extra MRI and MIBG examinations. In contrast, ctDNA was negative in up to eight consecutive samples at the end of treatment and during long-term follow-up of patients without relapse of the disease.

This study was retrospective and the results did not have an impact on clinical decisions. An important factor for prospective personalized ctDNA analysis is prompt access to the mutational spectrum in each individual tumor. Several large-scale efforts aiming to sequence tumor and germline DNA at time of diagnosis in children with cancer have been initiated, which paves the ground for patient-specific panel design (19-21). Nevertheless, implementation of ctDNA analysis with a personalized approach for clinical use is challenging and a streamlined process is required to achieve the results at a reasonable cost and in time for clinical decisions.

**Supplementary Figures**

**
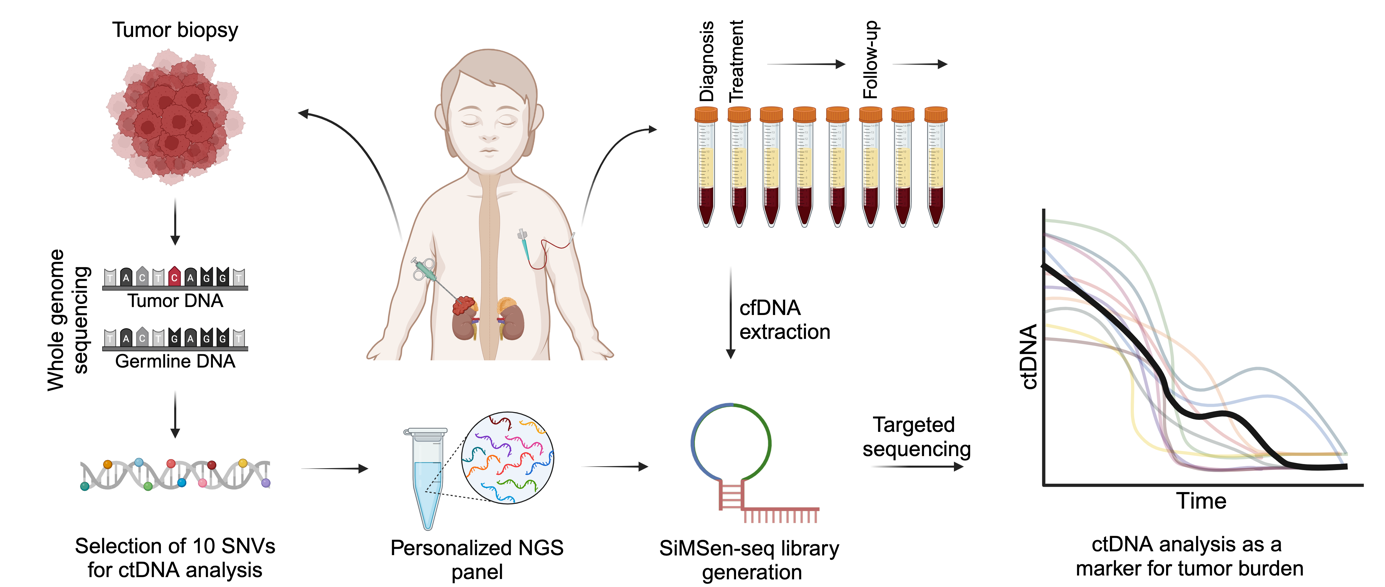
**

**Figure S1**. **Study design.** Tumor DNA from fresh frozen biopsy material and germline DNA from white blood cells were analyzed with whole genome sequencing. Ten SNVs per patient were selected based on tumor allele frequency in the primary tumor. A patient-specific multiplex sequencing panel was designed to cover the selected SNVs. Serial plasma samples were collected at diagnosis, and at regular intervals during and after treatment. cfDNA from all timepoints were analyzed with SiMSen-seq using the patient-specific multiplex panel. Finally, the levels of ctDNA were correlated to clinical parameters such as tumor volume and liquid biomarkers used in the clinic.

**
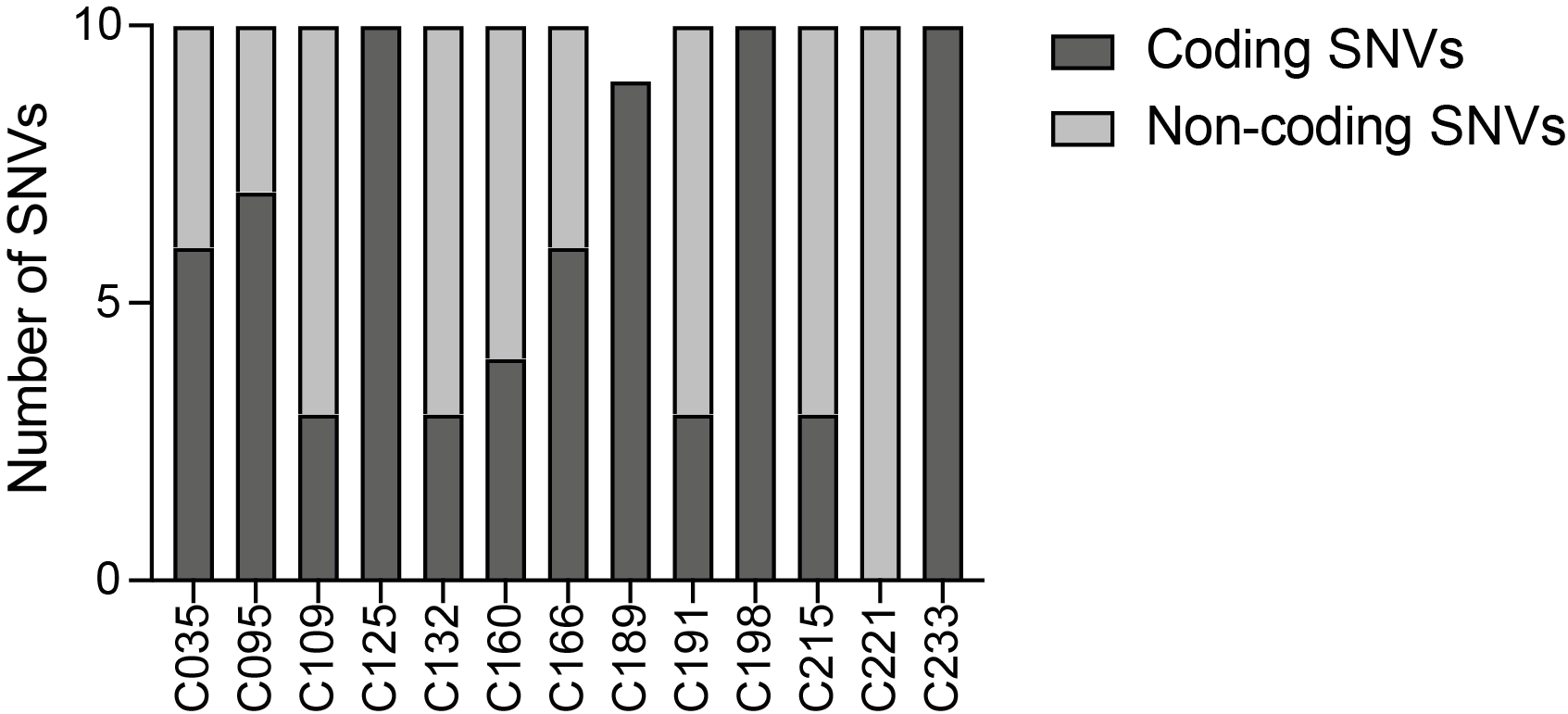
**

**Figure S2.** Number of coding and non-coding SNVs in each sequencing panel. One of the assays in panel C189 failed and generated no sequencing data, resulting in a panel with only nine SNVs.


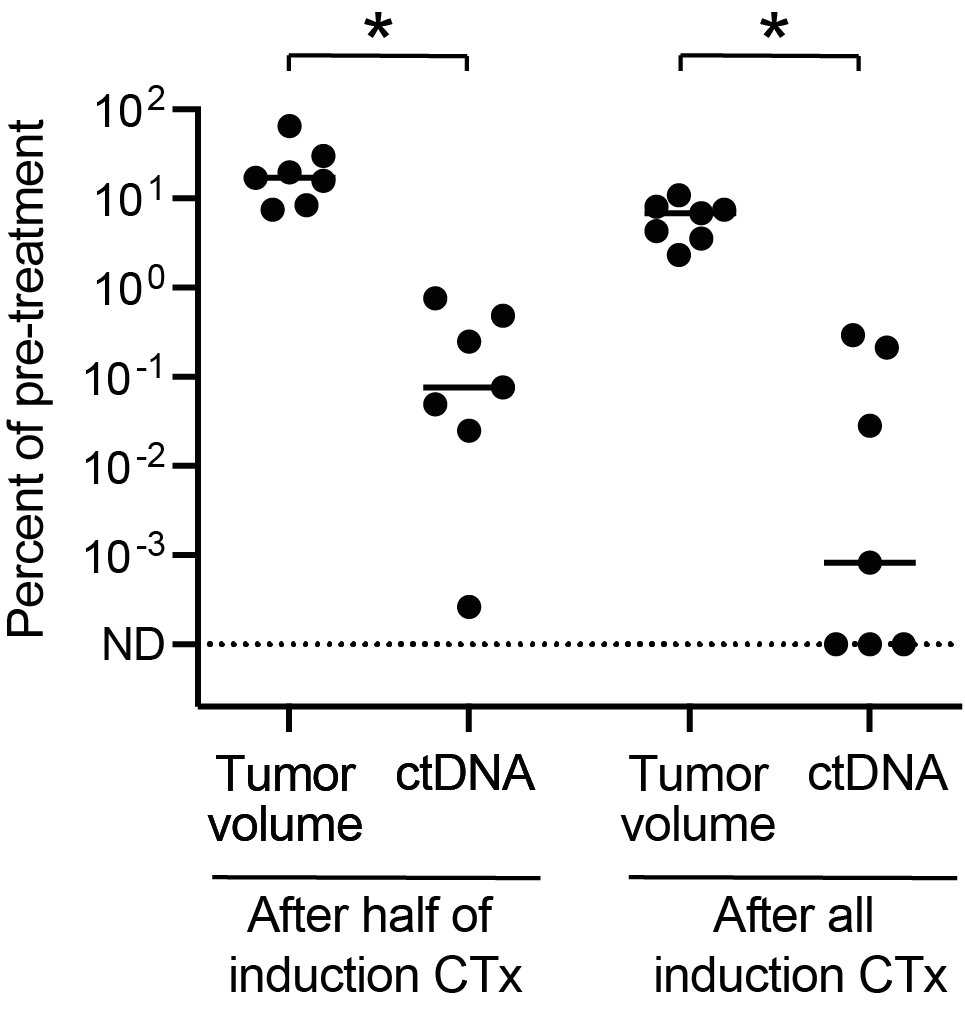


**Figure S3.** **Response to treatment as measured by tumor volume and ctDNA levels after half and all cycles of induction chemotherapy in patients with HR neuroblastoma.** Horizontal lines denote median values. Median remaining tumor burden after half of the induction CTx was 17% and 0.08% based on tumor volume and ctDNA, respectively. Median remaining tumor burden after all the induction CTx was 6.9% and 0.0008% based on tumor volume and ctDNA, respectively. *P* = 0.02 in both analyses, Wilcoxon matched-pairs signed rank test. CTx, chemotherapy.


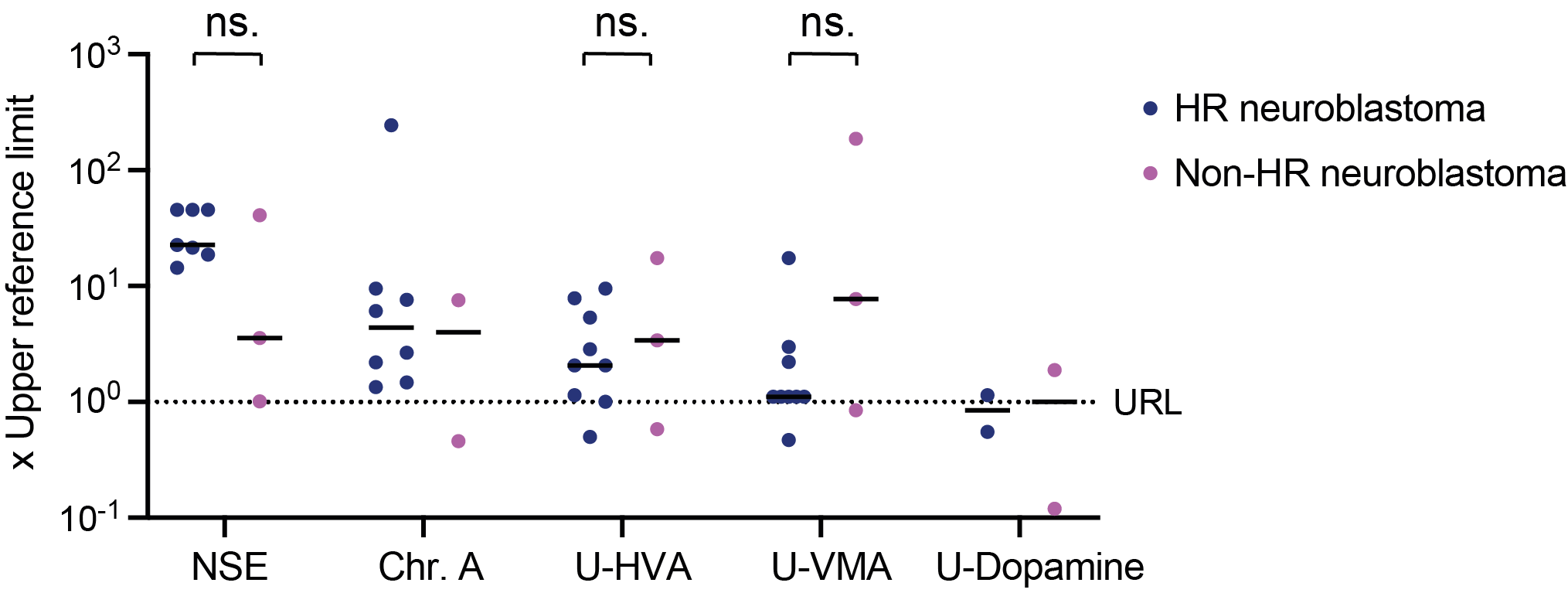


**Figure S4**. **Clinical tumor markers at time of diagnosis in HR vs. non-HR patients.** *P* (NSE) = 0.18; *P* (U-HVA) = 0.70; *P* (U-VMA) = 0.49, Mann Whitney test.


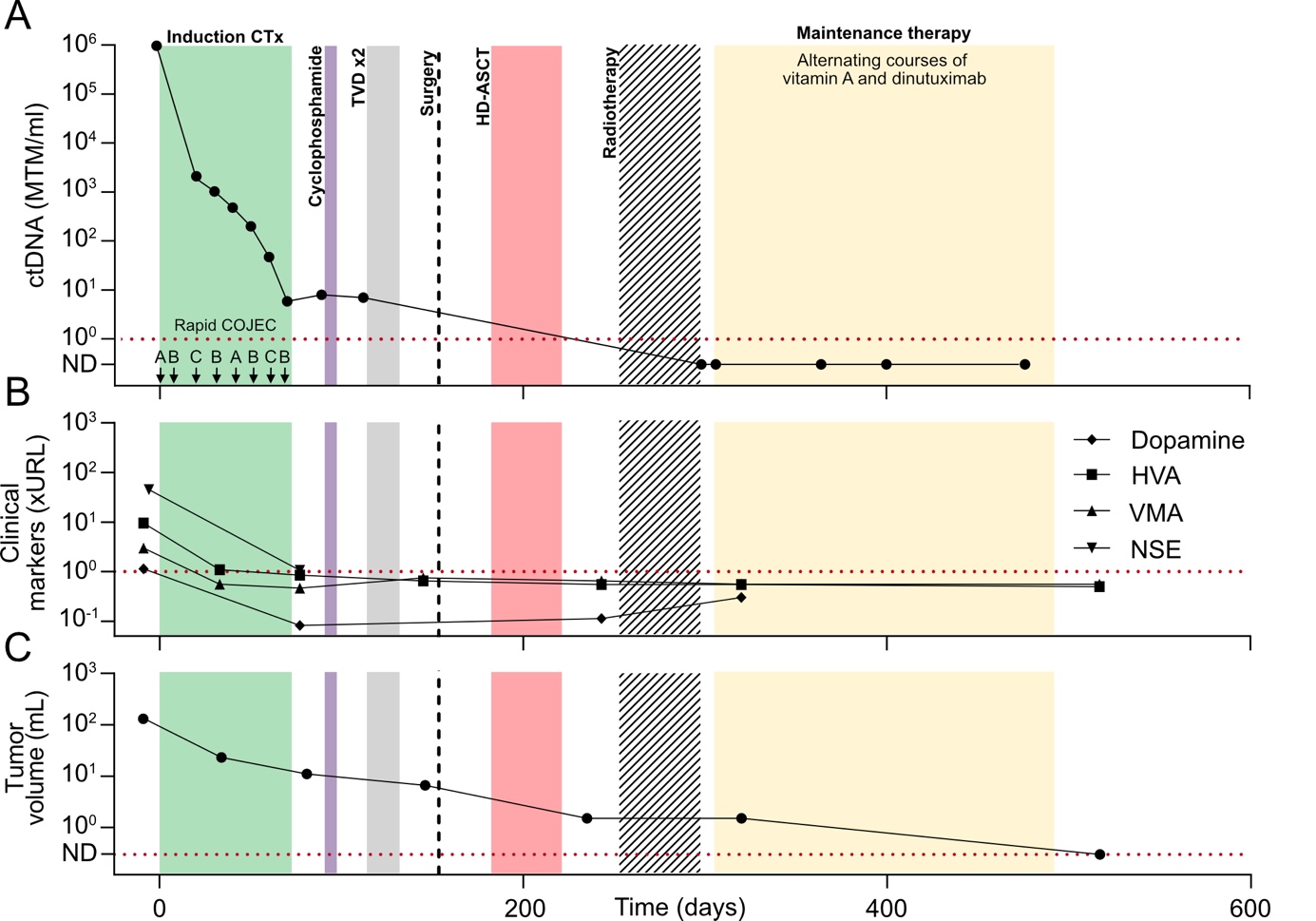


**Figure S5. Patient C095.** **(A)** Levels of ctDNA throughout the treatment. (**B)** Clinical tumor markers, normalized to upper reference limit (URL) as indicated by dashed line. **(C)** Approximated tumor volume based on CT (first two timepoints) and MRI (all other timepoints).


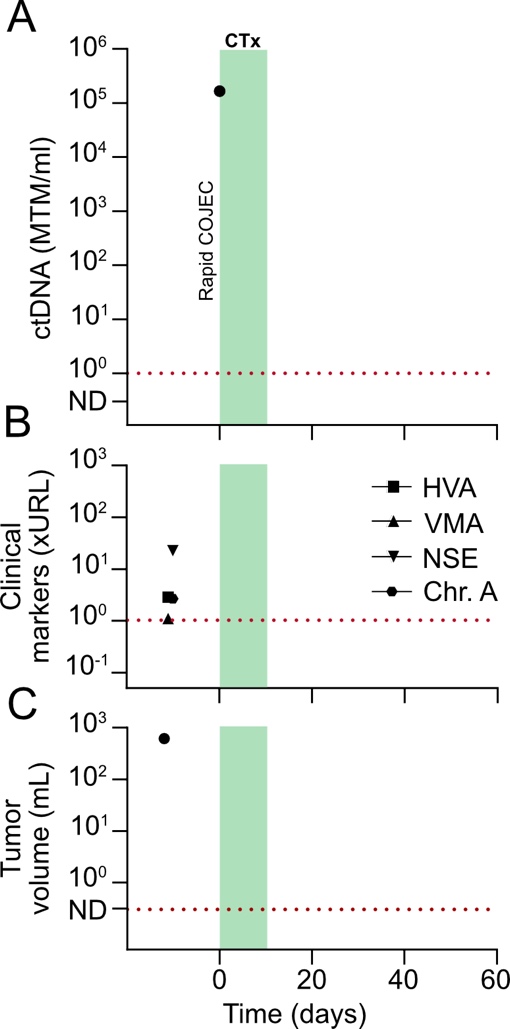


**Figure S6. Patient C166. (A)** Levels of ctDNA. **(B)** Clinical tumor markers, normalized to upper reference limit (URL) as indicated by dashed line. **(C)** Approximated tumor volume based on CT.


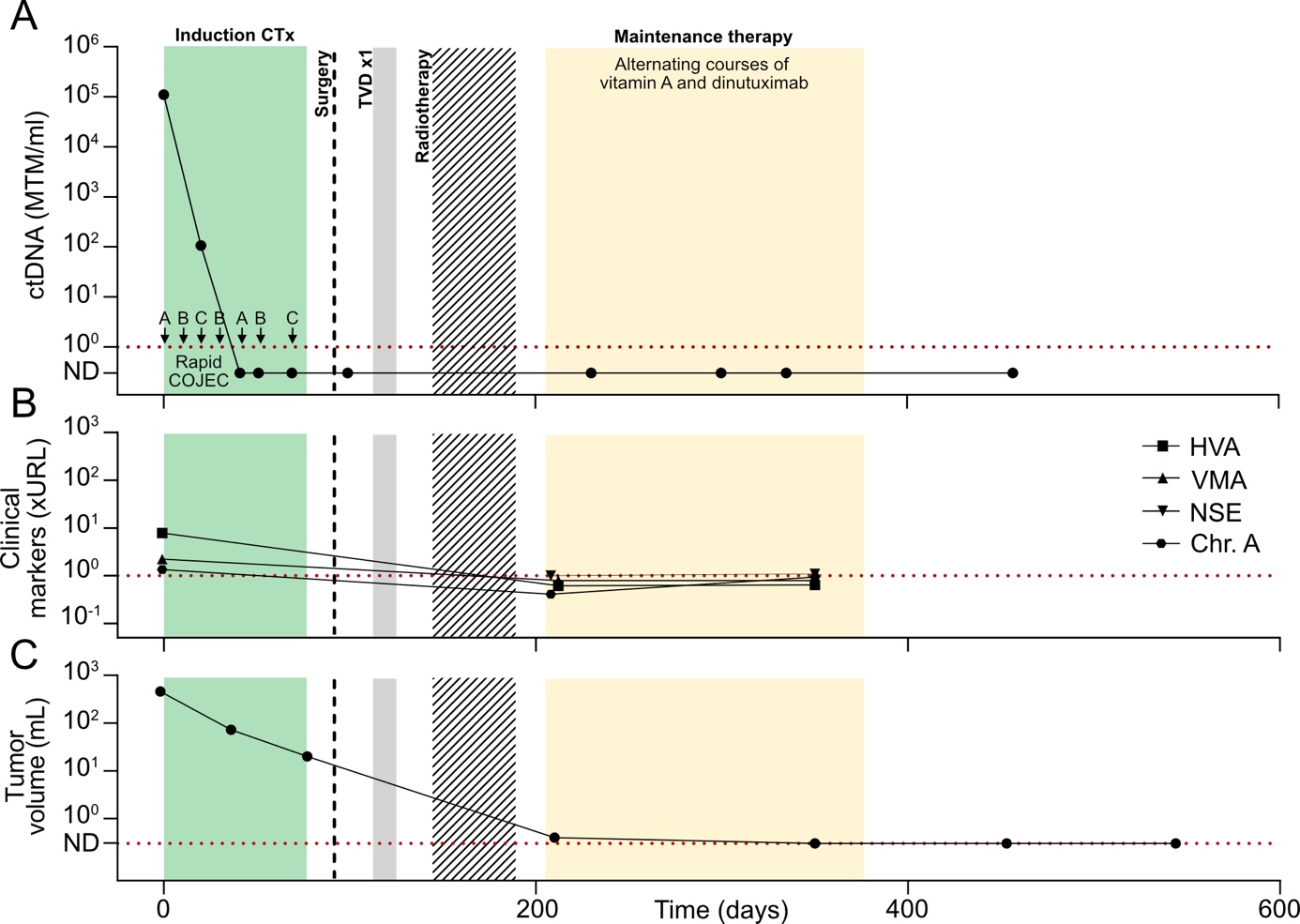


**Figure S7. Patient C191.** **(A)** Levels of ctDNA during and after treatment. **(B)** Clinical tumor markers, normalized to upper reference limit (URL) as indicated by dashed line. **(C)** Approximated tumor volume based on CT (timepoint five) or MRI (all other timepoints).


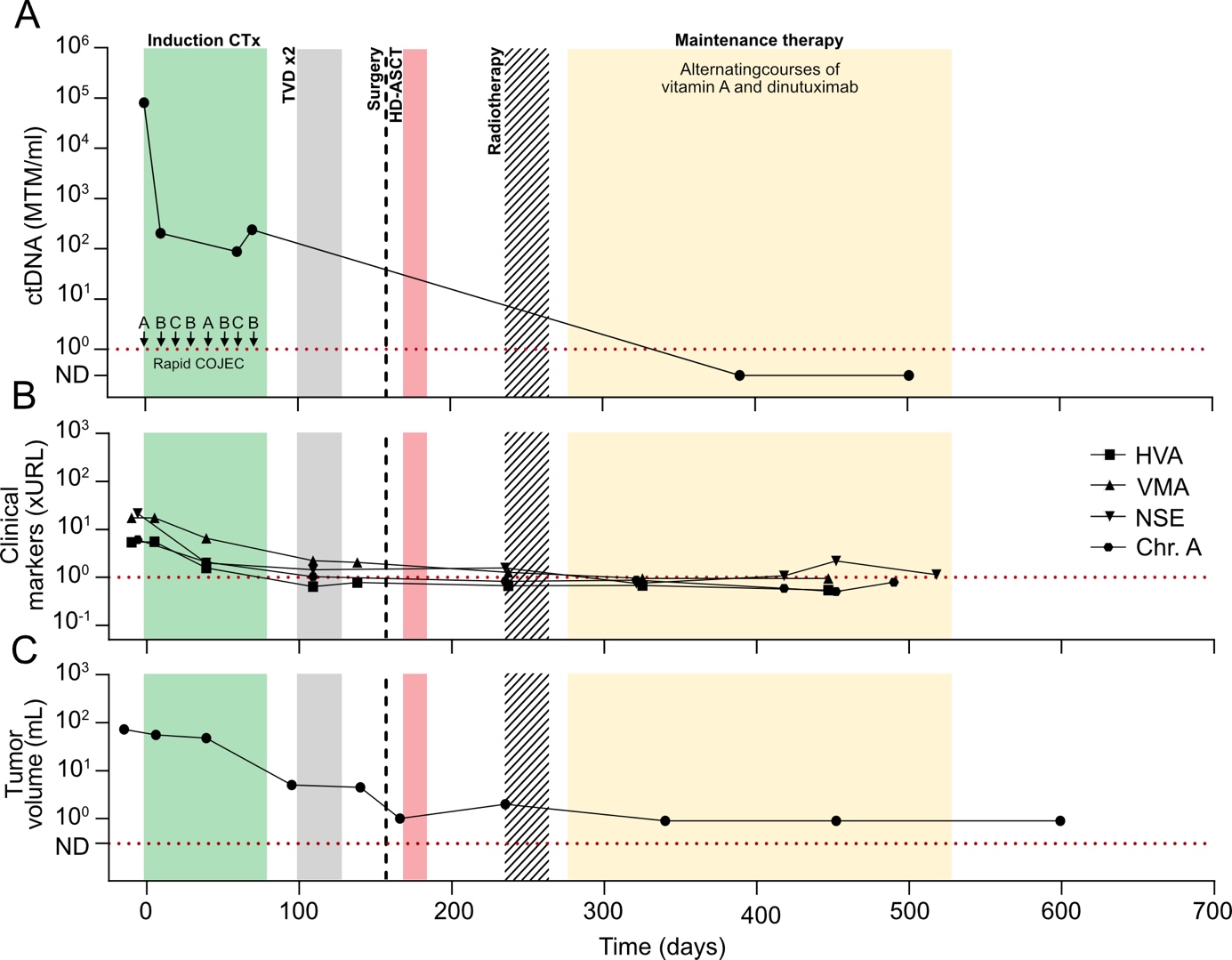


**Figure S8. Patient C198.** **(A)** Levels of ctDNA throughout the treatment. **(B)** Clinical tumor markers over time (normalized to upper reference limit (URL), as indicated by dashed line). **(C)** Approximated tumor volume based on CT (timepoint one, three, seven, eight, and ten) or MRI (all other timepoints).

**
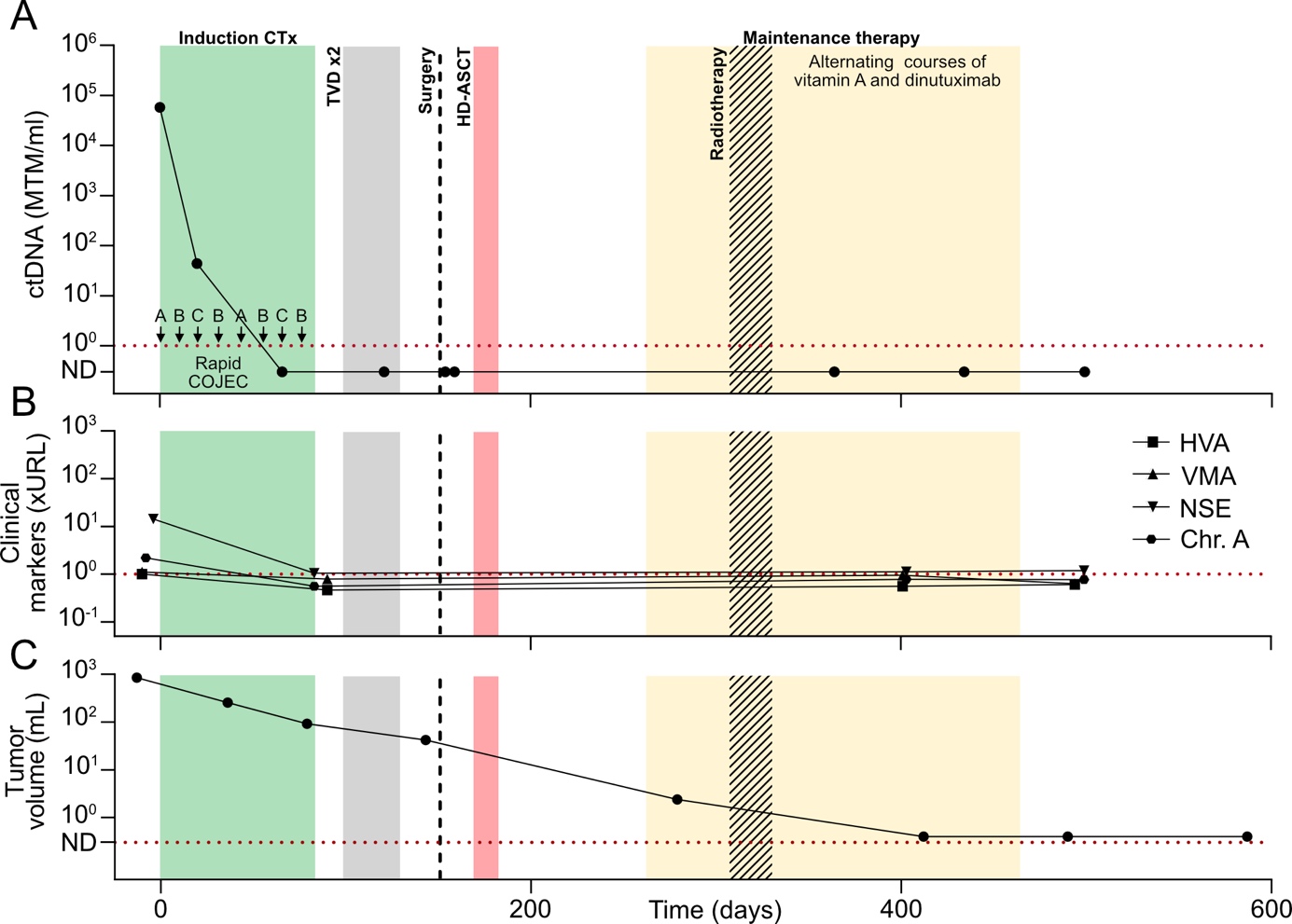
**

**Figure S9. Patient C215.** **(A**) Levels of ctDNA during and after treatment. **(B)** Clinical tumor markers over time, normalized to upper reference limit (URL) as indicated by dashed line. **(C)** Approximated tumor volume based on MRI (timepoint two and eight) or CT (all other timepoints).

**
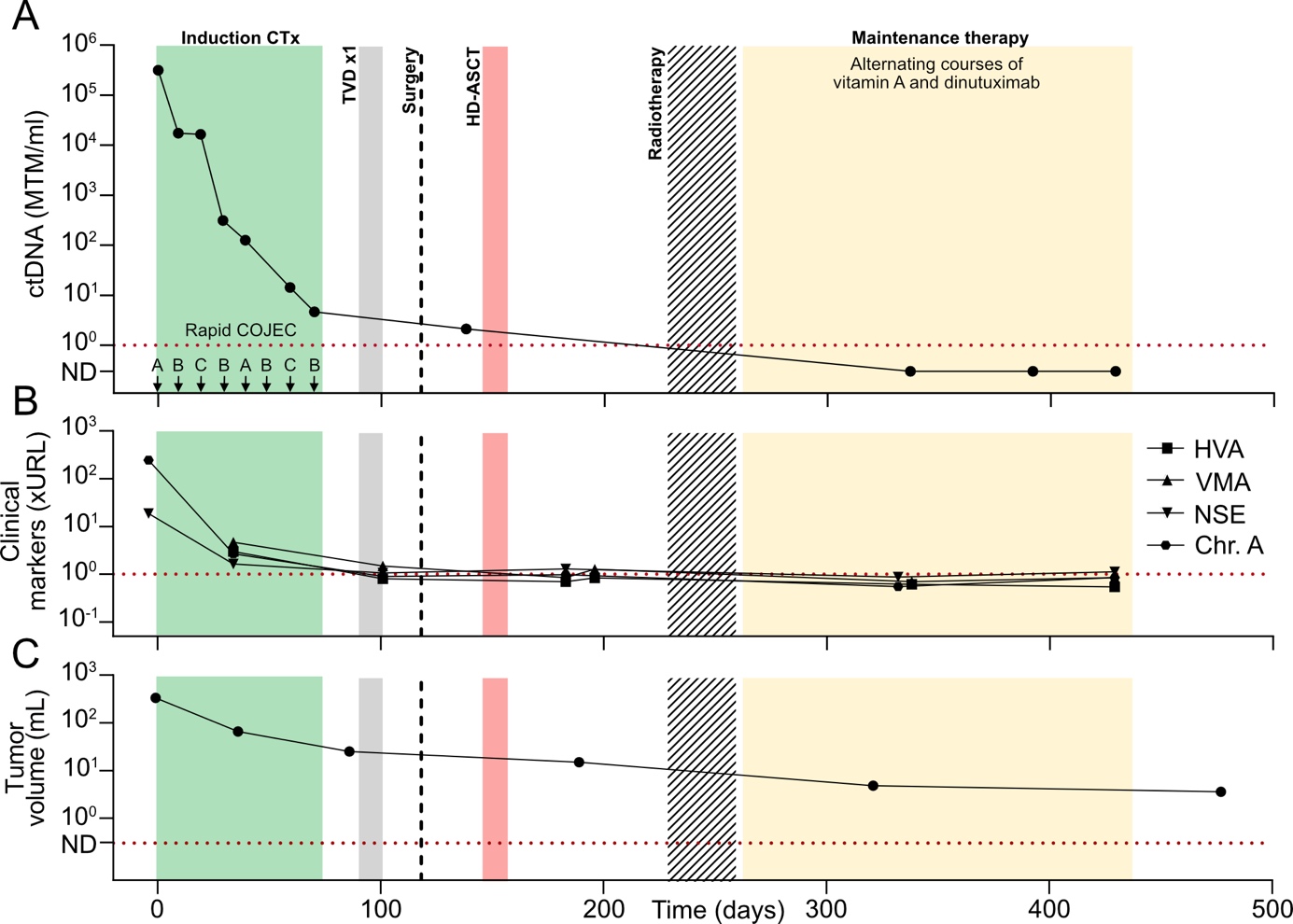
**

**Figure S10. Patient C233.** **(A)** Levels of ctDNA throughout the treatment. **(B)** Clinical tumor markers over time, normalized to upper reference limit (URL) as indicated by dashed line. **(C)** Approximated tumor volume based on MRI (timepoint one and six) or CT (all other timepoints).


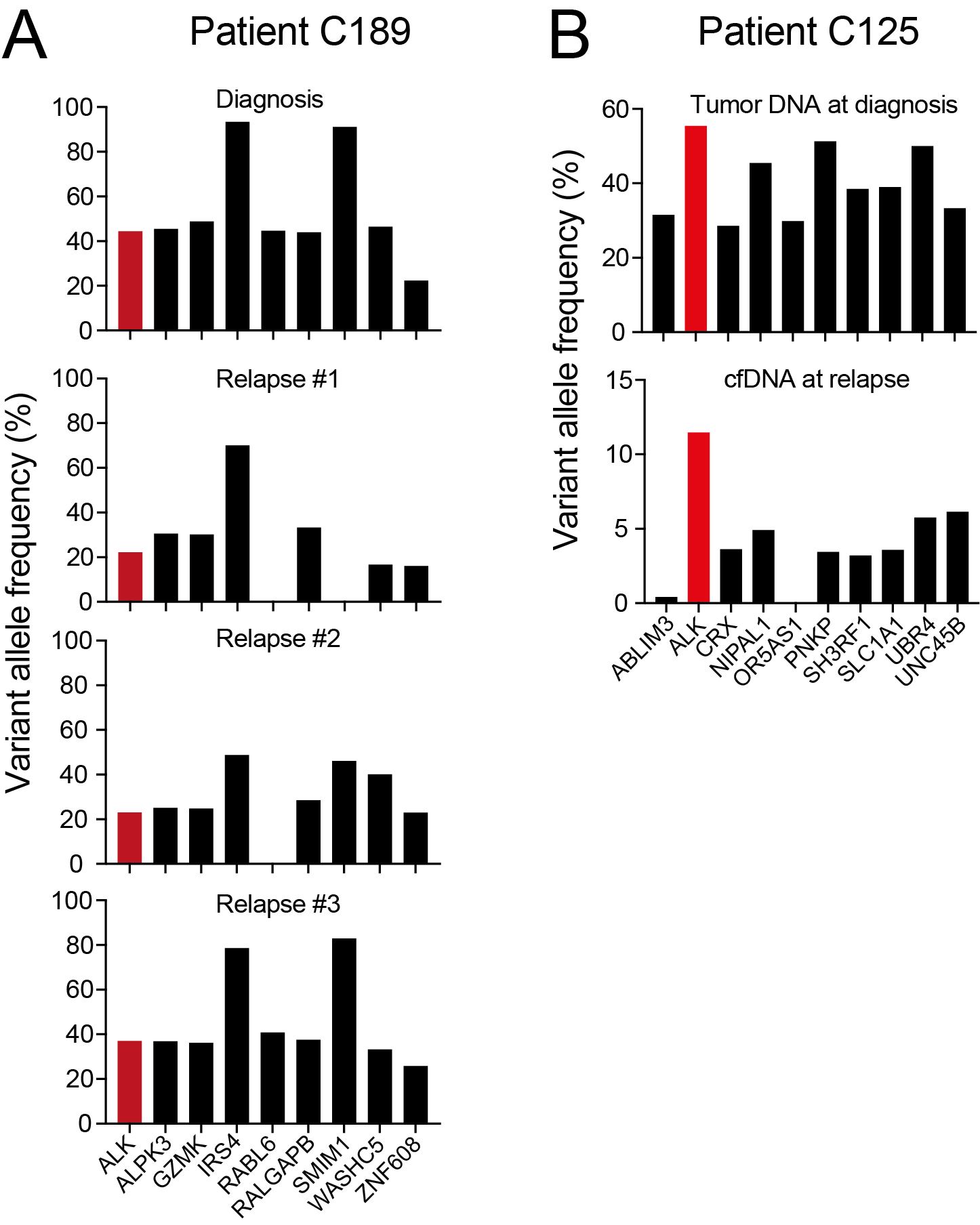


**Figure S11.** **(A)** Variant allele frequency in cfDNA for each individual SNV in the personalized sequencing panel at time of diagnosis and at the three relapses in patient C189. Red bars denote *ALK* p.F1174L. **(B)** Variant allele frequency for each SNV in the personalized sequencing panel in tumor biopsy DNA at time of diagnosis and in cfDNA at time of relapse. Red bars denote *ALK* p.R1275Q.

**Supplementary Tables**

| **Patient** | **Age^1^** | **Sex** | **Stage^2^** | **Risk^3^** | **1^st^ line treatment** | **Genetic alterations^4^** |
| --- | --- | --- | --- | --- | --- | --- |
| C035 | 4 m | m | M | HR | HR-NB (SIOPEN) | MNA, 1p del, 17q gain |
| C095 | 1y, 3m | f | M | HR | HR-NB (SIOPEN) | MNA, 1p del, 11q del, 17q gain, TERTr |
| C109 | 1y, 3m | f | L2 | LR | LINES group 3 | 2p gain, SCA |
| C125* | 9 y | f | L2 | HR | HR-NB (SIOPEN) | 1p del, ALK R1275Q |
| C132 | 15 y | f | L2 | IR | LINES group 7 | NCA + SCA Chr 16 |
| C160 | 3 y | m | M | HR | HR-NB (SIOPEN) | 1p del, 17q gain, *MYCC* amplification |
| C166 | 2 y | m | L2 | HR | HR-NB (SIOPEN) | MNA, 1p del, 2p gain |
| C189 | 2 y | m | M | HR | HR-NB (SIOPEN) | MNA, 1p del, 17q gain, ALK F1174L |
| C191 | 2 y | f | L2 | HR | HR-NB (SIOPEN) | MNA, 1p del, 17q gain |
| C198 | 2 y | f | M | HR | HR-NB (SIOPEN) | 1p del, 11q del |
| C215 | 3 y | m | M | HR | HR-NB (SIOPEN) | MNA, 1p del, whole 17 gain |
| C221 | 9 m | f | M | IR | LINES group 10 | 11q del, 2p gain, 17q gain |
| C233 | 6 y | m | M | HR | HR-NB (SIOPEN) | 1p del, 17q gain,11q del |

**Table S1. Patient characteristics.**  ^1^Age at time of diagnosis. ^2^Stage according to the International Neuroblastoma Risk Group Staging System (INRGSS). ^3^Risk group according to the international neuroblastoma risk group (INRG) classification. ^4^Selected genetic alterations. m, months; y, years; m, male; f, female; HR, high risk; IR, intermediate risk; LR, low risk; LINES, Low and intermediate risk neuroblastoma European study; HR-NB (SIOPEN), High-Risk Neuroblastoma Study 1.5 of SIOP-Europe; MNA, *MYCN* amplification; del, deletion; TERTr, TERT rearrangement; NCA, numerical chromosome alterations; SCA, segmental chromosome alterations. *Patient C125 was enrolled in the study at time of disease relapse.

| **Patient** | **Location** | **Tumor volume (ml)** | **MIBG** | **ctDNA^1^** | **Follow-up time** |
| --- | --- | --- | --- | --- | --- |
| C109 | Paravertebral | 34 | Not performed | Negative x1 | 56 months, NED |
| C132 | Paravertebral | 47 | Not perfomed | Negative x3 | 50 months, NED |
| C198 | Paravertebral | 0.5x2 | Positive | Negative x2 | 20 months, NED |
| C215 | Adrenal | 0.4 | Negative | Negative x3 | 18 months, NED |
| C233 | Paravertebral | 3.6 | Negative | Negative x3 | 17 months, NED |

**Table S2. Residual tumors at the end of treatment**.

Patients with residual tumors detectable on MRI or CT scan at first follow-up after the end of treatment. ^1^ctDNA results in samples collected during maintenance treatment or at follow-up visits after the end of treatment. NED, no evidence of disease.

**References:**

1. Monclair T, Brodeur GM, Ambros PF, Brisse HJ, Cecchetto G, Holmes K, et al. The International Neuroblastoma Risk Group (INRG) staging system: an INRG Task Force report. Journal of clinical oncology : official journal of the American Society of Clinical Oncology. 2009;27(2):298-303.

2. Ek T, Ibrahim RR, Vogt H, Georgantzi K, Träger C, Gaarder J, et al. Long-lasting response to lorlatinib in patients with ALK-driven relapsed or refractory neuroblastoma monitored with circulating tumor DNA analysis. Cancer Res Commun. 2024.

3. Johansson G, Andersson D, Filges S, Li J, Muth A, Godfrey TE, et al. Considerations and quality controls when analyzing cell-free tumor DNA. Biomolecular detection and quantification. 2019;17:100078.

4. Ye J, Coulouris G, Zaretskaya I, Cutcutache I, Rozen S, Madden TL. Primer-BLAST: a tool to design target-specific primers for polymerase chain reaction. BMC Bioinformatics. 2012;13:134.

5. Ståhlberg A, Krzyzanowski PM, Egyud M, Filges S, Stein L, Godfrey TE. Simple multiplexed PCR-based barcoding of DNA for ultrasensitive mutation detection by next-generation sequencing. Nature protocols. 2017;12(4):664-82.

6. Andersson D, Kebede FT, Escobar M, Österlund T, Ståhlberg A. Principles of digital sequencing using unique molecular identifiers. Molecular aspects of medicine. 2024;96:101253.

7. Osterlund T, Filges S, Johansson G, Stahlberg A. UMIErrorCorrect and UMIAnalyzer: Software for Consensus Read Generation, Error Correction, and Visualization Using Unique Molecular Identifiers. Clinical chemistry. 2022;68(11):1425-35.

8. Kahana-Edwin S, Cain LE, McCowage G, Darmanian A, Wright D, Mullins A, et al. Neuroblastoma Molecular Risk-Stratification of DNA Copy Number and ALK Genotyping via Cell-Free Circulating Tumor DNA Profiling. Cancers. 2021;13(13).

9. Combaret V, Iacono I, Bellini A, Bréjon S, Bernard V, Marabelle A, et al. Detection of tumor ALK status in neuroblastoma patients using peripheral blood. Cancer medicine. 2015;4(4):540-50.

10. Lodrini M, Graef J, Thole-Kliesch TM, Astrahantseff K, Sprüssel A, Grimaldi M, et al. Targeted Analysis of Cell-free Circulating Tumor DNA is Suitable for Early Relapse and Actionable Target Detection in Patients with Neuroblastoma. Clinical cancer research : an official journal of the American Association for Cancer Research. 2022;28(9):1809-20.

11. Kojima M, Hiyama E, Fukuba I, Yamaoka E, Ueda Y, Onitake Y, et al. Detection of MYCN amplification using blood plasma: noninvasive therapy evaluation and prediction of prognosis in neuroblastoma. Pediatric surgery international. 2013;29(11):1139-45.

12. Bosse KR, Giudice AM, Lane MV, McIntyre B, Schürch PM, Pascual-Pasto G, et al. Serial Profiling of Circulating Tumor DNA Identifies Dynamic Evolution of Clinically Actionable Genomic Alterations in High-Risk Neuroblastoma. Cancer discovery. 2022;12(12):2800-19.

13. Cimmino F, Lasorsa VA, Vetrella S, Iolascon A, Capasso M. A Targeted Gene Panel for Circulating Tumor DNA Sequencing in Neuroblastoma. Frontiers in oncology. 2020;10:596191.

14. Berko ER, Witek GM, Matkar S, Petrova ZO, Wu MA, Smith CM, et al. Circulating tumor DNA reveals mechanisms of lorlatinib resistance in patients with relapsed/refractory ALK-driven neuroblastoma. Nat Commun. 2023;14(1):2601.

15. Howie SR. Blood sample volumes in child health research: review of safe limits. Bulletin of the World Health Organization. 2011;89(1):46-53.

16. Bartolucci D, Montemurro L, Raieli S, Lampis S, Pession A, Hrelia P, et al. MYCN Impact on High-Risk Neuroblastoma: From Diagnosis and Prognosis to Targeted Treatment. Cancers. 2022;14(18).

17. www.foundationmedicine.com/sites/default/files/media/documents/2024-08/F1LCDx_Tech_Info_FDA_Label_Clear_RAL-0035%20v12.pdf

18. Traina TA, Norton L. Log-Kill Hypothesis. In: Schwab M, editor. Encyclopedia of Cancer. Berlin, Heidelberg: Springer Berlin Heidelberg; 2011. p. 2074-5.

19. Hodder A, Leiter SM, Kennedy J, Addy D, Ahmed M, Ajithkumar T, et al. Benefits for children with suspected cancer from routine whole-genome sequencing. Nat Med. 2024.

20. Church AJ, Corson LB, Kao PC, Imamovic-Tuco A, Reidy D, Doan D, et al. Molecular profiling identifies targeted therapy opportunities in pediatric solid cancer. Nat Med. 2022;28(8):1581-9.

21. Wadensten E, Wessman S, Abel F, Diaz De Ståhl T, Tesi B, Orsmark Pietras C, et al. Diagnostic Yield From a Nationwide Implementation of Precision Medicine for all Children With Cancer. JCO precision oncology. 2023;7:e2300039.
